# The Water Quality in Rio Highlights the Global Public Health Concern Over Untreated Sewage

**DOI:** 10.1289/EHP662

**Published:** 2016-10-01

**Authors:** Joseph N.S. Eisenberg, Jamie Bartram, Timothy J. Wade

**Affiliations:** 1 Department of Epidemiology, University of Michigan, Ann Arbor Michigan, USA; 2 The Water Institute, Department of Environmental Sciences and Engineering, University of North Carolina at Chapel Hill, Chapel Hill, North Carolina, USA; 3 United States Environmental Protection Agency, Chapel Hill, North Carolina, USA

## Abstract

Water quality issues in Rio have been widely publicized because of the 2016 Olympics. Recent concerns about polluted waters that athletes may be exposed to highlights the conditions that more than a billion people globally are exposed to daily. Despite these unhealthy conditions, much is unknown about the risks and exposure pathways associated with bathing in or drinking untreated or partially treated sewage. Beyond acute illness, we are learning more about the chronic sequelae that arise from repeated exposure to pathogens found in sewage. Additionally, we do not know enough about how to measure water quality, especially in developing countries. A consequence of these knowledge gaps is that data from developed countries are used to guide public health approaches in low- and middle-income settings. More data that are locally specific are needed to inform guidelines for improving sanitation and water quality in Rio and other cities in developing countries.

## Introduction

From the spread of the Zika virus, to the polluted waters in Rio de Janeiro, media reports have highlighted health concerns in Brazil. An editorial in the *New York Times* (Cox 2016), along with other media reports of high levels of sewage contamination in Rio’s recreational waters, caused wide-ranging concerns about the safety of sailing, rowing, and other open-water events at the 2016 Olympic Games (Brooks and Barchfield 2015). Such concerns seemed well grounded, because in August 2015, members of the U.S. rowing team reported stomach illness following a trial competition on a Rio lake that the team doctor suspected was due to water pollution (Mazloumsaki and Brocchetto 2015). For both Zika and water quality, media attention has focused on athletes and the role of the International Olympic Committee. However, exposure goes beyond athletes to everyone who uses water, including tourists and most importantly local residents. That much of Rio’s human sewage goes untreated is in no way unusual for urban environments, especially in low- and middle-income countries (LMICs). It is estimated that 1.5 billion people worldwide have sewage connections with no treatment (Baum et al. 2013). Even in higher-income countries where sewage is often treated to a high degree, environmental contamination from sewage occurs when treatment is bypassed, for example, during storm water overflows, which are frequent in some settings (Sato et al. 2013).

## Monitoring Recreational and Drinking Water

The news coverage about Rio highlights the need for a better understanding of exposures and risks associated with untreated or partially treated sewage that exposes billions to dangerously high levels of pathogens worldwide. Sewage-contaminated waters contain a wide range of potentially pathogenic bacteria, protozoa, and viruses. The World Health Organization (WHO 2003) and the U.S. Environmental Protection Agency (U.S. EPA 2012) provide general recommendations for monitoring drinking water and recreational waters, largely based on data from developed countries in temperate climates. These include simple as well as targeted tests for bacteria commonly found in human feces, such as *Escherichia coli* or *Enterococcus*, which are used to gauge the level of contamination and to infer potential health risks (Wade et al. 2003). However, the use of these bacteria as markers has been questioned in tropical environments because of a concern that they tend to grow or have more prolonged survival in warm environments than in the climates the recommendations are based on (Byappanahalli and Fujioka 2004; Stewart et al. 2008).

In a rare study in a tropical developing country, a recent analysis found that the bacterial indicators used by the WHO and other agencies were, in fact, a good measure of human risk for locations like Rio, because many of the popular beaches in Rio and in other cities in developing countries worldwide are often directly affected by untreated sewage (Lamparelli et al. 2015). In this prospective cohort study, Lamparelli et al. (2015) conducted a study of beach goers (*N* = 16, 637) who were exposed to sewage-contaminated waters on five beaches in São Paulo, Brazil. The research team recruited families during a 1-month period (9 January through 6 February 1999) who were then followed up through a telephone interview. The evidence provided by Lamparelli et al. (2015) that currently used bacterial indicators are appropriate in tropical beaches under the influence of untreated sewage, where their validity has been questioned, has important implications for regulation and practice worldwide.

However, recent media reports from Rio, as well as at least one scientific study, have suggested that not only are standard bacterial indicators inadequate, but that there may be a need to test for specific viral pathogens in Rio waters (Brooks 2015; Victoria et al. 2014). In our view, the proposed focus on testing for a relatively narrow range of viral pathogens is misguided, because the most critical factor in preventing health risks under conditions of untreated-sewage discharges is to identify the degree of sewage contamination rather than the presence of a small subset of the wide range of pathogens of potential concern. To identify the degree of sewage contamination, standard tests for bacterial or viral indicator organisms, which are commonly found in high densities in sewage are just as good, cheaper, and more reliable in settings where resources are limited (WHO 2003). These tests can usefully be combined with sanitary surveys to understand the source and nature of discharges to the water bodies and to inform decision-making about remediation.

Under some circumstances, there may be a need for additional testing beyond fecal indicator organisms to better characterize water quality and to understand human health risks. For example, in settings where exposure to fecal contamination is low because of advanced sewage treatment, standard bacterial indicators could die off more rapidly than many viral and protozoan pathogens (Rose et al. 1996). In tropical environments with advanced sewage treatment, where water temperatures are higher, persistence or possible growth of bacterial indicators may also call for alternative measures, supplemental measures, or both. Under these conditions, alternative monitoring approaches and indicators that are *a*) better indicators of viral contamination, *b*) more resistant to advanced treatment technologies, and *c*) more sensitive at lower levels of pathogens, may be warranted (Savichtcheva and Okabe 2006). Also, in cases where sites are affected not by sewage point sources but by diffuse and variable non-point sources, such as where latrines and septic systems provide sanitation services to human populations, standard bacterial indicators may not be sufficient to identify pathogen contamination. Under these circumstances it may be important to determine the specific source or type of fecal contamination using source-specific genetic markers.

## Exposure to Untreated Sewage is a Global Problem

Exposure to human feces, including untreated sewage and the pathogens associated with it, is a global problem and extends beyond recreational waters and the 2016 Olympics. The risks of acute and chronic disease attributable to exposure to human excreta are largely unknown. Over the past decades, there has been a dramatic increase in the number of people using improved drinking water sources (such as village wells with hand pumps) worldwide, and the Millennium Development Goal (MDG) objective to halve the proportion of those using unimproved drinking water sources was achieved ahead of the deadline. In contrast, the number of people without access to improved sanitation is high (Baum et al. 2013), and the sanitation objective fell short by a large margin as the MDGs drew to a close in 2015 (Fuller et al. 2016; WHO/UNICEF 2015). We believe that the general lack of data on the associated risks is cause for concern—especially with the global population growth and the even more rapid growth in the number of households, which are outstripping the ability to treat and manage human feces (Bartram et al. 2012).

## Conclusion

This situation illustrates the complexity of testing and monitoring sewage in the environment. Standard bacterial indicators were predictive of gastrointestinal illness in one of the few recreational water studies that measured health risks in a tropical setting where sewage is largely untreated (Lamparelli et al. 2015). On the surface, it may seem beneficial and informative to test for viral pathogens, but the additional expertise and resources to conduct these tests may divert resources from routine monitoring without substantially increasing our understanding of the risks or improving health outcomes. The global trends toward urbanization have intensified our need for water and have created new emerging hazards such as antimicrobial resistance. We should, therefore, view the water quality issues highlighted by the media attention to the Rio Olympics as a wake-up call. The Rio condition has also reminded us that the development of the associated sewerage and sewage-treatment infrastructures is a very costly, long-term enterprise that cannot be activated for rapid effect. It is urgent that we invest in improving our management, including the treatment of human excreta and sewage and the development of tools to track and optimize these efforts.
